# Association between Infancy BMI Peak and Body Composition and Blood Pressure at Age 5–6 Years

**DOI:** 10.1371/journal.pone.0080517

**Published:** 2013-12-04

**Authors:** Michel H. P. Hof, Tanja G. M. Vrijkotte, Marieke L. A. de Hoog, Manon van Eijsden, Aeilko H. Zwinderman

**Affiliations:** 1 Department of Clinical Epidemiology, Bioinformatics, and Biostatistics, Academic Medical Center—University of Amsterdam, Amsterdam, The Netherlands; 2 Department of Public Health, Academic Medical Center—University of Amsterdam, Amsterdam, The Netherlands; 3 Department of Epidemiology, Documentation and Health Promotion, Public Health Service of Amsterdam (GGD), Amsterdam, The Netherlands; Peking Union Medical College, China

## Abstract

**Introduction:**

The development of overweight is often measured with the body mass index (BMI). During childhood the BMI curve has two characteristic points: the adiposity rebound at 6 years and the BMI peak at 9 months of age. In this study, the associations between the BMI peak and body composition measures and blood pressure at age 5–6 years were investigated.

**Methods:**

Measurements from the Amsterdam Born Children and their Development (ABCD) study were available for this study. Blood pressure (systolic and diastolic) and body composition measures (BMI, waist-to-height ratio, fat percentage) were gathered during a health check at about 6 years of age (n = 2822). All children had multiple BMI measurements between the 0–4 years of age. For boys and girls separately, child-specific BMI peaks were extracted from mixed effect models. Associations between the estimated BMI peak and the health check measurements were analysed with linear models. In addition, we investigated the potential use of the BMI at 9 months as a surrogate measure for the magnitude of the BMI peak.

**Results:**

After correction for the confounding effect of fetal growth, both timing and magnitude of the BMI peak were significantly and positively associated (p<0.001) with all body composition measures at the age of 5–6 years. The BMI peak showed no direct association with blood pressure at the age 5–6 year, but was mediated by the current BMI. The correlation between the magnitude of the BMI peak and BMI at 9 months was approximately 0.93 and similar associations with the measures at 5–6 years were found.

**Conclusion:**

The magnitude of the BMI peak was associated with body composition measures at 5–6 years of age. Moreover, the BMI at 9 months could be used as surrogate measure for the magnitude of the BMI peak.

## Introduction

While the incidence of childhood obesity appears to have plateaued in recent years in developing and developed countries, prevalence is still concerningly high [1]–[3]. In addition, the prevalence of childhood hypertension has increased rapidly in the last decades [4], [5]. Both phenomena come with a large number of adverse health events and social problems during childhood [4]–[8] but there is also evidence suggesting that both obesity [9]–[11] and hypertension [12]–[14] have a high probability of tracking into adulthood. Because hypertension is the leading risk factor for cardiovascular disease [9], [15] and is highly related to body mass index (BMI) status [16], detection of early childhood growth patterns that might predict the risk of obesity or hypertension has received much interest [17]–[19].

For the average child, the BMI changes substantially with age in early childhood [20]–[22], resulting in two characteristic points on the change-curve that can be identified. From birth till around the age of nine months the BMI increases to a maximum, referred to as the BMI peak [23]. After this peak the BMI decreases to a minimum around the age of 6 years, which is often referred to as the adiposity rebound [24], [25], before the BMI increases again.

Although consistent evidence has been found that an early adiposity rebound is related to both an increased probability of obesity [24]–[27] and higher blood pressure [28] at later age, little research has been performed to determine the relevance of the BMI peak in the tracking mechanism of obesity and blood pressure. Silverwood et al. [23] found that both the timing of the BMI peak and the magnitude of the BMI peak were positively associated with later BMI z-scores between the ages of 5 and 13 years. In addition, Mook-Kanamori et al. [29] discovered that a higher BMI peak significantly increases the probability of obesity. A recent study from Wen et al. [30] showed that , contrary to the timing of the BMI peak 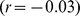, the magnitude of the BMI peak is strongly positively correlated to the magnitude of the adiposity rebound 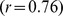. This study shows that especially the magnitude of the BMI peak has an important role in the tracking mechanism of BMI.

The BMI peak is an interesting point in the BMI change curve of a child because current literature suggests that it is predictive for later BMI and correlates with the location of the adiposity rebound. In addition, because the BMI peak is located around 9 months of age [26], it can be earlier detected than the adiposity rebound which occurs at approximately 6 years of age. However, there are some problems with using the BMI peak to measure early childhood growth patterns. The BMI peak is often not observed because it requires a lot of BMI measurements around the age of 9 months. In addition, Silverwood et al. [23] and Wen et al. [30] showed that the timing of the BMI peak is only weakly correlated to later BMI. Therefore, a cross-sectional BMI measurement at 9 months of age, which is considerably easier to obtain than estimating the BMI peak, might contain the same information as the BMI peak in the tracking mechanism of BMI.

Previous studies [23], [29], [30] only investigated BMI as the outcome. However, BMI is a combination of fat mass and fat free mass and from growth studies it is known that accelerated postnatal growth results in fat deposition with a preference for abdominal fat [31]. Therefore it is interesting to determine whether the BMI peak is associated with fat mass and waist to height ratio. Moreover, the three previously published studies did not include blood pressure as an outcome measure. Many studies showed that postnatal growth, independent from prenatal growth, leads to higher blood pressure [32], [33].

To further explore whether the magnitude and the timing of the BMI peak are predictive for later body composition and blood pressure, we measured the association between the BMI peak and the body composition measures BMI, waist to height ratio, fat percentage, in addition to systolic and diastolic blood pressure at about 6 years of age using growth data from the prospective Amsterdam Born Children and their Development (ABCD) cohort study. We also investigated whether the BMI at 9 months of age could be used as a surrogate measure for the magnitude of the BMI peak.

## Materials and Methods

### Study population

The ABCD study initially focused on the relation between lifestyle characteristics of the mother during pregnancy and the development of the child, with specific interest in ethnic differences [34]. Approval was obtained from the medical ethics committee of the Academic Medical Center - University of Amsterdam and the registration committee of the municipal board of the city of Amsterdam, the Netherlands. All participants gave written informed consent for themselves and their children. Between January 2003 and March 2004, all pregnant women living in Amsterdam (n = 12373) were invited to participate at their first antenatal visit to an obstetric caregiver. After this visit a language-specific questionnaire was sent to the pregnant women's home address covering sociodemographic data, obstetric history, lifestyle, dietary habits and psychological factors. The questionnaire was returned by 8266 women. In addition to the questionnaire, 6575 mothers gave permission to collect data from the Amsterdam Youth Health Care registration. This registration contains growth data, date of delivery, gender, birth weight, and gestational age of all children. [34].

#### BMI measurements

In total, we were able to extract BMI measurements from 5686 singleton ABCD children (2831 boys and 2855 girls) between the ages of 0–6 years from the Amsterdam Youth Health Care registration. Because we wanted to focus on the association between the BMI peak and later measurements of healthy children, the data from 331 preterm born children (pregnancy duration shorter than 37 weeks) were excluded from the analysis. Furthermore, we decided that each child should have at least three BMI observations between the ages of 0–4 years to accurately model the child specific BMI curve. Three BMI measurements were required because with less than three measurements, the individual BMI growth curve needed to derive the BMI peak is almost completely based on the population average. Therefore 212 children with fewer than three measurements were excluded from the data. This meant that 5143 children (2543 boys and 2600 girls) with three or more valid BMI measurements were available for the current study.

#### Follow-up measurements at 5–6 years of age

**Figure 1:**
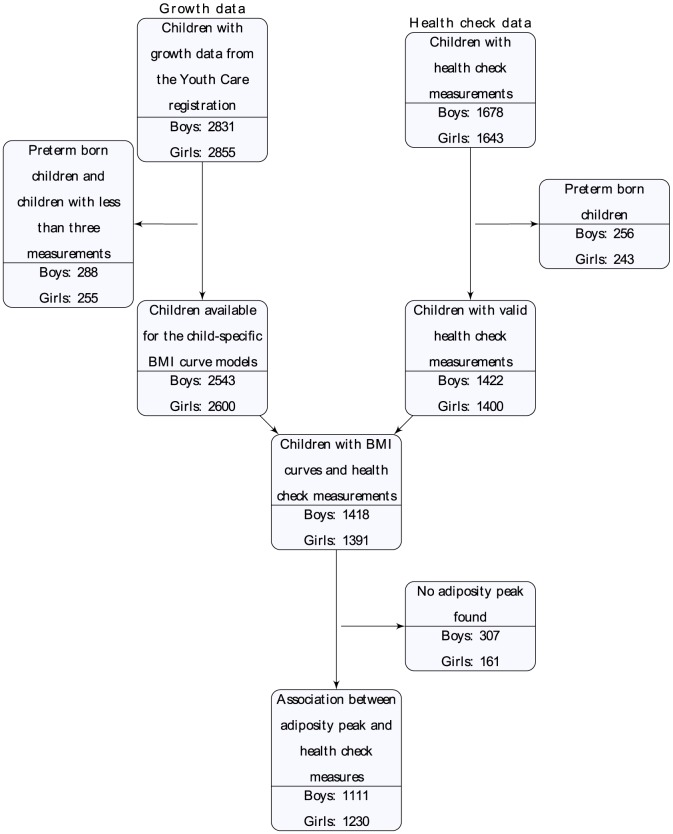
Number of children available for this study.

During the summer of 2008, around the fifth birthday of the ABCD children, the mothers were sent a questionnaire in which they were asked for permission regarding participation of their child in the ABCD health check. From the 6161 mothers with a traceable address, 3321 children were measured at the ABCD health check at the age of 5–6 years. Similar to the BMI measurements, we excluded the preterm born children. In total, 2822 children (1422 boys and 1400 girls) with the ABCD health check measurements were available for the analyses (figure 1).

During the health check, among other characteristics, height, weight, waist circumference, fat percentage, and blood pressure were measured. Height was determined to the nearest millimeter using a Leicester portable height measure (Seca, Hamburg, Germany) and weight to the nearest 100 gram using a Marsden MS-4102 weighing scale (Oxfordshire, United Kingdom). Waist circumference was measured to the nearest millimeter midway between the costal border and iliac crest, using a Seca measuring tap. In addition, fat percentage was derived from two bioelectrical impedance analysis measurements using the Bodystat 1500 MDD system (Bodystat Inc, Douglas, United Kingdom). Blood pressure was measured in sitting position twice using the Omron 705 IT (Omron Healthcare Inc, Bannockburn, IL, USA) with a small cuff (arm circumference 17–22 cm) on the non-dominant arm. After the child was acclimatized by letting him/her sit at a table for a couple of minutes, the blood pressure was measured two times. When either the systolic or diastolic pressure differed more than 10 mmHg between the two measurements, a third measurement was taken (502 children). In the current study, the average of the two measurements closest together was used. More detail on the measurements during the ABCD health check can be found in the article by van Dijk et al. [35].

### Data Analysis

The analysis of the association between the BMI peak and the measurements at the ages 5–6 years was performed with the following steps. Firstly, child-specific BMI growth curves were constructed from all available growth data up to age 4 years from which the BMI peaks were extracted. Secondly, the associations between the BMI peak and the measures at the ages 5–6 years were calculated using linear regression models. After this analysis, the predictive value of the BMI peak was evaluated with the explained variance of the measures at the ages 5–6 years.

For the BMI at 9 months we calculated the associations between the BMI peak and the measures at the ages 5–6 years with linear regression models. In addition, we evaluated the explained variances of all models and compared them to the explained variances of the models with the BMI peak. After these analyses, the relation between the BMI at 9 months and the BMI was investigated.

#### Child specific BMI trajectories and BMI peaks

On average, children were measured 11.5 times in the first four years of life with a standard deviation of 2.6. Growth models were fitted to the data for boys and girls separately because of the known gender differences in growth observed in the ABCD study [22]. Subject-specific BMI curves were created with semi-parametric mixed effect models. Linear mixed effects models were fitted to the data with natural spline functions for both the fixed and random effects to capture the non-linear trend in the BMI trajectories. Notice that the mixed effect models are able to capture the child specific growth curve in age regions with many points. In other regions with fewer observations, the growth curve is more influenced by the population average. The formulation of the models is described in detail in Rupert et al. [36].

For the natural splines in the mixed effect models the number of knots and their placement needed to be determined. The location of the knots was fixed by placing them at the quantiles of the distribution of age. For instance, a natural spline with seven knots has the knots placed at the (0/6)*^th^*, (1/6)*^th^*, (2/6)*^th^*, …, (5/6)*^th^*, (6/6)*^th^* quantile of the age distribution. The number of knots in the natural spline determines the smoothness of the relation between BMI and age. Using too many knots will lead to overfitting the data and too few knots to a linear fit. The optimum number of knots in the natural splines was determined with the Bayesian information criterion (BIC) [37] and was respectively 6 and 6 for the fixed and random effects in both the model for boys and the model for girls. The BMI peak, described by its magnitude (in kg m^−2^) and timing (in months), is located at the point at which the derivative of the child-specific BMI curve equals zero. Children with no or more than one identified BMI peak were excluded from the analyses.

#### Association with the 5–6 years measurements

We estimated the association between the BMI peak and body composition measures (BMI, waist to height ratio as waist circumference in cm./height in cm., and fat percentage), and blood pressure measures (sitting systolic and sitting diastolic blood pressure) gathered at the health check between 5–6 years of age. Linear regression models were fitted to the data to measure the association between the BMI peak characteristics (magnitude and timing) and the outcomes at the health check (body composition and blood measures).

To measure the association between the BMI peak and the body composition measures at 5–6 years of age, we fitted a model with the following covariates

- BMI peak characteristics (magnitude and timing)
- birth weight
- pregnancy duration
- age at the health check

In addition to the BMI peak characteristics, birth weight and pregnancy duration were added to the analysis as linear predictors. Fetal growth (expressed by birth weight and pregnancy duration) is related to the obesity status at later age [38] and an increased birth weight is associated with lower systolic blood pressure at later ages [15]. Because not all children were measured at exactly the same age at the health check, we also added the age at the health check to the model as a linear predictor because all outcome measures are likely to change with age.

For the blood pressure measures, three models were fitted to the data because current blood pressure is strongly related to the current height [39] and current BMI status [40]. Therefore, we fitted the following models to measure whether the association between BMI peak and blood pressure was mediated by current height or BMI.

Model 1 Model 2 Model 3

• BMI peak characteristics (magnitude and timing) • BMI peak characteristics (magnitude and timing) • BMI peak characteristics (magnitude and timing)• birth weight • birth weight • birth weight • pregnancy duration • pregnancy duration • pregnancy duration • age at the health check • age at the health check • age at the health check • current height • current height • current BMI

#### Explained variance of the outcomes at 5–6 years

To measure the relevance of the magnitude and timing of the BMI peak, we investigated the explained variance of all the models that were used to estimate the associations with the 5–6 years measurements. For the body composition measures, each model was first fitted without the BMI peak characteristics. We then added the timing of the BMI peak and the magnitude of the BMI peak sequentially to the linear regression models to measure the increase in explained variance. A similar procedure was performed for the three blood pressure models (see previous section).

#### BMI at 9 months of age

Because the BMI of children was often not measured at exactly 9 months of age, we took the BMI measurement between 8–10 months that was closest to 9 months as a proxy for the BMI at 9 months. Similarly to the BMI peak analyses, we calculated the associations between the BMI at 9 months and the measures at 5–6 years of age. Furthermore, we investigated the explained variance of the regression models with the BMI at 9 months (instead of the BMI peak characteristics).

## Results

### ABCD cohort

In total, 2809 (1418 boys and 1391 girls) children had both ABCD health check measurements between the age of 5–6 years and three or more BMI measurements between 0–4 years. From this group, 468 children (307 boys and 161 girls) had no or multiple peaks in their BMI curve and therefore 2341 children (1111 boys and 1230 girls) were available for stage two of the analysis (figure 1). For the analysis of BMI at 9 months, we included 721 boys and 801 girls who had BMI measurements between 8–10 months of age.

**Figure 2:**
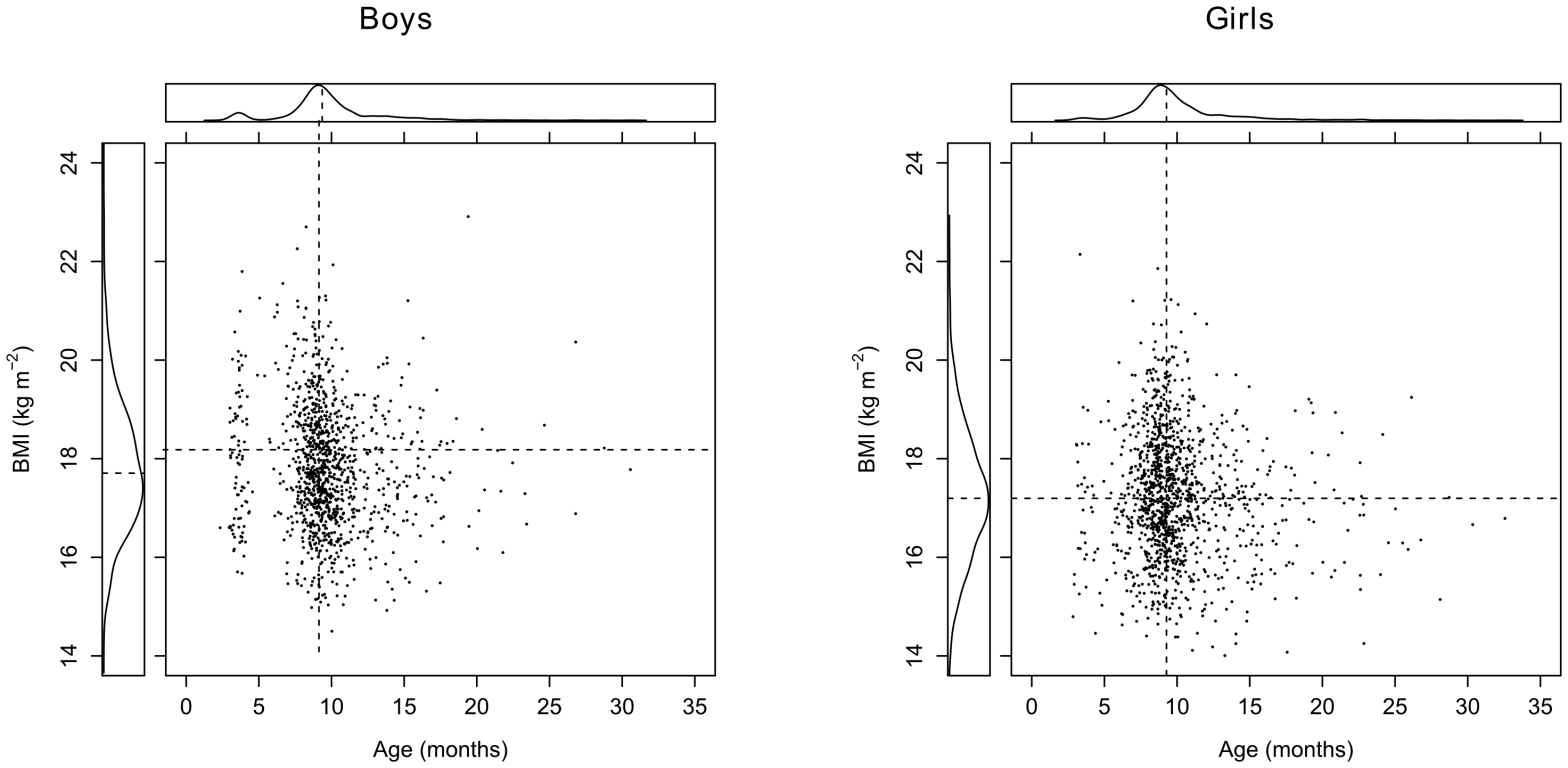
Distribution of the estimated BMI peaks. The dotted lines represent the median BMI peak characteristics. Note that both the height and age at the BMI peak are approximately symmetrically distributed for both genders.

The median timing of the BMI peak was 9.36 (95% CI: 3.44–17.49) months for boys and 9.27 (95% CI: 4.30–19.64) months for girls. The median magnitude of the BMI peak was 17.71 (95% CI: 15.50–20.55) kg m^−2^ for boys and 17.20 (95% CI: 14.95–19.79) kg m^−2^ for girls (figure 2). Both the timing and magnitude of the BMI peak were approximately symmetrically distributed around their median value. Most BMI peaks were observed between the ages of 6 and 18 months. Timing and magnitude of the BMI peak were weakly negatively correlated (r = −0.08 for boys and r = −0.09 for girls). With these low correlations, the associations between the health measurements at 5–6 years and the BMI peak characteristics do not change drastically when the peak characteristics are analysed separately or simultaneously, and therefore the results from the univariate analyses are not reported.

For the 2341 children with an identified BMI peak, the distribution of the measured characteristics at the 5–6 year health check are summarized in table 1. Between boys and girls no significant (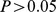) differences were found in body composition and blood pressure measures. Mean age at the health check was 69 months. Characteristics were available for almost all children. Children were born after a mean pregnancy duration of 280 days, with a standard deviation of 8 days.

**Table 1:** Baseline characteristics.

Birth outcome				
	Boys (n = 1111)		Girls (n = 1230)	
Characteristic	Observations	mean (s.d.)	Observations	mean (s.d.)
Pregnancy Duration (days)	1111	281 (8.4)	1230	280 (8.4)
Birth Weight (gram)	1111	3587 (504)	1230	3419 (462)
BMI at 9 months of age (kg m^−2^)	721	17.7 (1.3)	801	17.0 (1.4)

BMI peak characteristics				
	Boys (n = 1111)		Girls (n = 1230)	
Characteristic	Observations	median (2.5%–97.5%)	Observations	median (2.5%–97.5%)
Timing (months)	1111	9.36 (3.44–17.49)	1230	9.27 (4.30–19.64)
Magnitude (kg m^−2^)	1111	17.71 (15.50–20.55)	1230	17.20 (14.95–19.79)

Health check measurements at 5–6 years				
	Boys (n = 1111)		Girls (n = 1230)	
Characteristic	Observations	mean (s.d.)	Observations	mean (s.d.)
Age (months)	1111	68.8 (5.9)	1230	68.9 (6.1)
Height (cm)	1085	117.0 (5.8)	1220	116.4 (5.7)
Weight (kg)	1084	21.3 (3.10)	1219	21.0 (3.45)
BMI (kg m^−2^)	1084	15.5 (1.35)	1219	15.4 (1.54)
Waist to height ratio	1084	0.45 (0.03)	1218	0.45 (0.03)
Fat Percentage (%)	1065	19.41 (5.79)	1196	21.33 (6.53)
Systolic blood pressure (mm Hg)	1072	98.8 (8.5)	1199	98.2 (8.4)
Diastolic blood pressure (mm Hg)	1072	58.8 (8.3)	1199	60.2 (8.0)

Characteristics of the 2341 children with valid BMI peaks and health check measurements.

### Association between BMI peaks and 5–6 year health check measurements

#### Body composition measures

Table 2 describes the relation between the BMI peak characteristics and the body composition measures at 5–6 years of age. For boys and girls similar trends between the BMI peak and body composition measures were found. Generally, a higher and later peak led to a significant (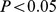) increase in all body composition measures at 5–6 years of age.

**Table 2:** Associations between BMI peak and body composition measures at 5–6 years of age.

		Boys			Girls		
Outcome	Covariate	Estimate	Std. Error	P-value	Estimate	Std. Error	P-value
BMI (kg m^−2^)	Intercept	15.511	0.033	—	15.442	0.036	—
	Timing of the BMI peak (months)	0.071	0.010	<0.001	0.076	0.010	<0.001
	Magnitude of the BMI peak (kg m^−2^)	0.622	0.027	<0.001	0.697	0.030	<0.001
	Birth weight (kg)	0.093	0.076	0.220	0.064	0.086	0.460
	Pregnancy duration (days)	0.005	0.004	0.234	−0.003	0.005	0.515
Waist to	Intercept	45.216	0.074	—	44.749	0.079	—
Height	Timing of the BMI peak (months)	0.081	0.023	<0.001	0.118	0.023	<0.001
ratio×100	Magnitude of the BMI peak (kg m^−2^)	0.893	0.063	<0.001	0.945	0.065	<0.001
	Birth weight (kg)	−0.474	0.173	0.006	−0.368	0.190	0.054
	Pregnancy duration (days)	0.015	0.01	0.136	0.008	0.010	0.432
Fat	Intercept	19.430	0.161	—	21.347	0.169	—
percentage (%)	Timing of the BMI peak (months)	0.187	0.050	<0.001	0.215	0.049	<0.001
	Magnitude of the BMI peak (kg m^−2^)	1.597	0.136	<0.001	1.877	0.139	<0.001
	Birth weight (kg)	1.038	0.375	0.006	0.166	0.408	0.683
	Pregnancy duration (days)	0.004	0.021	0.864	−0.023	0.022	0.295

Estimated regression parameters, standard errors, and P-values for the models estimating the relation between the BMI peak, pregnancy duration and the body composition outcomes at the health check. All covariates were centralized around their mean value. The age at outcome measurement was centred around 69 months (coefficients not reported in table).

With each added month to the timing of the BMI peak, the BMI measured at 5–6 years of age increased respectively 0.07 kg m^−2^ and 0.08 kg m^−2^ for boys and girls. In addition, an increase of the magnitude of the BMI peak with one kg m^−2^ raised the BMI at 5–6 years with 0.6 kg m^−2^ for boys and 0.7 kg m^−2^ for girls. Both birth weight and pregnancy duration were not significantly associated with BMI at 5–6 years (adjusted for magnitude and timing of the BMI peak).

One month added to the timing of the BMI peak increased the waist to height ratio with 0.8×10^−3^ for boys and 1.2×10^−3^ for girls. Adding one kg m^−2^ to the magnitude of the BMI peak gave an increase of 9.0×10^−3^ for both genders. Birth weight was negatively associated with the waist to height ratio for both genders, although the association was not significant for girls. Pregnancy duration was not significantly associated with the waist to height ratio.

Fat percentage at 5–6 years of age increased 0.2% for both genders with each month added to the timing of the BMI peak. For each kg m^−2^ added to the magnitude of the BMI peak, fat percentage increased with 1.6% and 1.9% for respectively boys and girls. Birth weight was significantly positively associated with fat percentage for boys, but not for girls. In addition, pregnancy duration was not significantly associated with fat percentage for both genders.

#### Blood pressure measures

In tables 3 and 4 the associations between the BMI peak and the blood pressure measures at 5–6 years of age are summarized. The associations with the timing of the BMI peak were non-significant in all models for both boys and girls. For the magnitude of the BMI peak different associations with blood pressure at 5–6 years were found for boys and girls.

**Table 3:** Associations between BMI peak and systolic blood pressure at 5–6 years of age.

Systolic blood pressure							
		Boys			Girls		
Outcome	Covariate	Estimate	Std. Error	P-value	Estimate	Std. Error	P-value
Model 1	Intercept	98.913	0.250	—	98.234	0.236	—
	Timing of the BMI peak (months)	0.043	0.078	0.576	0.087	0.068	0.202
	Magnitude of the BMI peak (kg m^−2^)	0.401	0.212	0.059	0.764	0.195	<0.001
	Birth weight (kg)	−1.713	0.584	0.003	−0.896	0.567	0.114
	Pregnancy duration (days)	0.022	0.033	0.500	0.006	0.030	0.837
Model 2	Intercept	98.879	0.247	—	98.225	0.233	—
	Timing of the BMI peak (months)	−0.001	0.077	0.992	0.072	0.067	0.282
	Magnitude of the BMI peak (kg m^−2^)	0.378	0.209	0.071	0.640	0.193	0.001
	Birth weight (kg)	−2.640	0.598	<0.001	−1.561	0.572	0.006
	Pregnancy duration (days)	0.034	0.033	0.297	0.019	0.030	0.531
	Height at outcome measurement (cm)	0.307	0.054	<0.001	0.291	0.052	<0.001
Model 3	Intercept	98.861	0.241	—	98.232	0.231	—
	Timing of the BMI peak (months)	−0.109	0.077	0.155	−0.002	0.068	0.981
	Magnitude of the BMI peak (kg m^−2^)	−0.613	0.248	0.013	−0.032	0.23	0.890
	Birth weight (kg)	−2.685	0.584	<0.001	−1.482	0.566	0.009
	Pregnancy duration (days)	0.025	0.032	0.433	0.022	0.030	0.466
	Height at outcome measurement (cm)	0.273	0.053	<0.001	0.235	0.052	<0.001
	BMI at outcome measurement (kg m^−2^)	1.604	0.226	<0.001	1.012	0.193	<0.001

Estimated regression parameters, standard errors, and P-values for the linear multivariate regression models describing the relation between the BMI peak, pregnancy duration and systolic blood pressure outcomes at the health check. All covariates were centralized around their mean value. In addition, the age at outcome measurement was centered around 69 months (coefficients not reported in table).

**Table 4:** Associations between BMI peak and diastolic blood pressure at 5–6 years of age.

Diastolic blood pressure							
		Boys			Girls		
Outcome	Covariate	Estimate	Std. Error	P-value	Estimate	Std. Error	P-value
Model 1	Intercept	58.835	0.247	—	60.176	0.228	—
	Timing of the BMI peak (months)	−0.052	0.077	0.498	0.013	0.066	0.839
	Magnitude of the BMI peak (kg m^−2^)	0.204	0.209	0.330	0.538	0.187	0.004
	Birth weight (kg)	−1.572	0.576	0.006	−1.748	0.546	0.001
	Pregnancy duration (days)	0.069	0.033	0.034	0.048	0.029	0.101
Model 2	Intercept	58.826	0.247	—	60.171	0.227	—
	Timing of the BMI peak (months)	−0.064	0.077	0.405	0.005	0.065	0.935
	Magnitude of the BMI peak (kg m^−2^)	0.198	0.209	0.345	0.469	0.188	0.013
	Birth weight (kg)	−1.826	0.598	0.002	−2.116	0.556	<0.001
	Pregnancy duration (days)	0.072	0.033	0.027	0.055	0.029	0.06
	Height at outcome measurement (cm)	0.084	0.054	0.116	0.161	0.05	0.001
Model 3	Intercept	58.814	0.245	—	60.177	0.225	—
	Timing of the BMI peak (months)	−0.134	0.078	0.086	−0.057	0.066	0.388
	Magnitude of the BMI peak (kg m^−2^)	−0.438	0.251	0.082	−0.100	0.224	0.657
	Birth weight (kg)	−1.855	0.593	0.002	−2.049	0.552	<0.001
	Pregnancy duration (days)	0.067	0.032	0.040	0.057	0.029	0.048
	Height at outcome measurement (cm)	0.063	0.053	0.242	0.113	0.051	0.027
	BMI at outcome measurement (kg m^−2^)	1.029	0.230	<0.001	0.856	0.188	<0.001

Estimated regression parameters, standard errors, and P-values for the linear multivariate regression models describing the relation between the BMI peak, pregnancy duration and diastolic blood pressure outcomes at the health check. All covariates were centralized around their mean value. In addition, the age at outcome measurement was centred around 69 months (coefficients not reported in table).

For boys, non-significant associations were found between the magnitude of the BMI peak and both systolic and diastolic blood pressure measures at 5–6 years of age. These associations remained when the current height (i.e. measured at the health check) was added to the model. When both current BMI and current height were added as extra covariates to the regression model, the magnitude of the BMI peak showed a weak but significant association with systolic blood pressure. Each kg m^−2^ added to the magnitude of the BMI peak decreased systolic blood pressure with 0.6 mm Hg. Results for diastolic blood pressure remained non-significant.

For girls, significant associations were found between the magnitude of the BMI peak and both systolic and diastolic blood pressure measures at 5–6 years. An increase of one kg m^−2^ of the magnitude of the BMI peak increased the systolic blood pressure with 0.8 mm Hg and the diastolic blood pressure with 0.5 mm Hg. The magnitude of the BMI peak remained significantly associated with an increase of both blood pressure measures when current height was added to the model. However, when both current BMI and current height were added to the model, the magnitude of the BMI peak was no longer associated with both blood pressure measures. This meant that the association between the magnitude of the BMI peak and blood pressure was completely mediated by the current BMI.

#### Explained variance of the health check outcomes at 5–6 years

The explained variance in the models that estimated the association between the BMI peak and later health check outcomes at 5–6 years are summarized in table 5. When the magnitude of the peak was added to the model, the explained variance for the BMI at 5–6 years was 34% and 32% for respectively boys and girls. When timing was added to the model, the explained variance only slightly increased to 37% and 35% respectively. Lower explained variances were found for waist for height ratio, where the explained variance was 19% for boys and 16% for girls when the magnitude was added to the model. Similar results were found for fat percentage at 5–6 years (17% for boys and 19% for girls). The explained variances were only slightly increased when timing was added to the models (approximately 1% more).

**Table 5:** Explained variance BMI peak and BMI at 9 months.

		Boys (n = 1111)					Girls (n = 1230)				
		None	BMI peak characteristics			BMI at	None	Added BMI peak characteristics			BMI at
						9 months					9 months
Outcome			Timing	Magnitude	Timing and			Timing	Magnitude	Timing and	
					magnitude					magnitude	
BMI		5.8	7.2	34.2	37.1	30.5	3.6	5.0	32.1	34.9	24.2
Waist for height ratio		4.2	4.5	18.7	19.6	15.2	2.6	3.6	16.0	17.8	14.0
Fat percentage		6.7	7.3	16.8	17.9	14.9	6.9	7.6	18.6	19.9	14.6
Systolic blood	Model 1	6.8	6.8	7.1	7.1	8.6	4.7	4.7	5.8	6.0	4.0
pressure	Model 2	9.6	9.6	9.9	9.9	11.6	7.5	7.6	8.3	8.4	6.1
	Model 3	13.4	13.5	13.8	14.0	14.0	10.5	10.5	10.5	10.5	9.5
Diastolic blood	Model 1	4.4	4.5	4.5	4.5	3.6	3.3	3.3	4.0	4.0	2.7
pressure	Model 2	4.6	4.7	4.7	4.8	4.1	4.3	4.3	4.8	4.8	3.1
	Model 3	6.1	6.3	6.3	6.5	5.2	6.4	6.4	6.4	6.4	5.7

Explained variance (in %) for all models used to calculate the association between the BMI peak or BMI at 9 months and the outcomes at 5–6 years of age. Note that the model without BMI peak characteristics or BMI at 9 months is the model denoted as none which only as the covariates birth weight , pregnancy duration, and age at the health check in the model. The other models included the BMI peak characteristics or the BMI at 9 months in addition to these characteristics.

For the blood pressure at 5–6 years, adding the magnitude and timing of the BMI peak could not explain more variance than the model with current height, current BMI, pregnancy duration, and birth weight (model 3). This model explained about 13% for boys and 11% for girls of the variance in systolic blood pressure at 5–6 years. For diastolic blood pressure at 5–6 years the explained variance was about 6% for both genders.

#### BMI at 9 months of age

The associations between BMI at 9 months of age and the body composition measures and blood pressure measurements at 5–6 years are summarized in tables S1, S2 and S3. Compared to the magnitude of the BMI peak, the associations with the 5–6 year body composition measures were slightly lower for BMI at 9 months but still (highly) significant. With an increase of one kg m^−2^ of the BMI at 9 months, the 5–6 years BMI increased 0.50 kg m^−2^ for boys and 0.49 kg m^−2^ for girls, the weight to height ratio at 5–6 years increased 0.72 for boys and 0.69 for girls and fat percentage at the age of 5–6 years increased 1.28% for boys and 1.40% for girls.

The explained variances in the models with BMI at 9 months were lower than the models with the magnitude of the BMI peak. For instance, for BMI at 5–6 years the explained variance was 31% and 24% for respectively boys and girls with the model that included BMI at 9 months of age (table 5). This was lower than the 34% for boys and 32% for girls that could be explained with the model that included the magnitude of the BMI peak.

**Figure 3:**
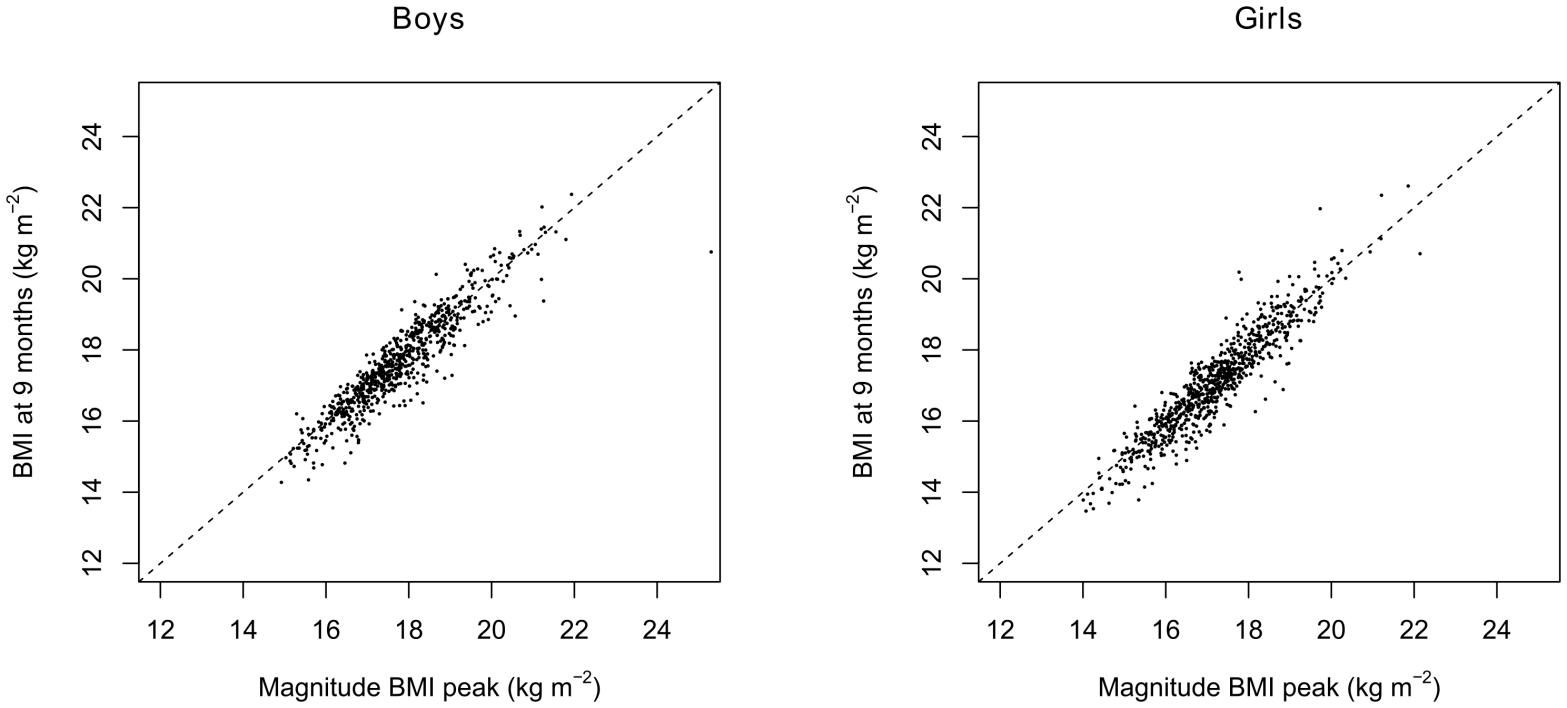
BMI at 9 months and the magnitude of the BMI peak. The correlation between the BMI at 9 months of age and the magnitude of the BMI peak was 0.925 for both genders.

The BMI at 9 months of age was strongly correlated with the magnitude of the BMI peak and was 0.925 for both genders. This strong correlation can also be seen in figure 3.

## Discussion

### Main findings

We investigated associations between the BMI peak, located at approximately 9 months of age, and body composition measures and blood pressure at the age of 5–6 years. Generally, a higher and later BMI peak resulted in higher BMI, higher fat percentage, and higher waist to height ratio in boys and girls at the age of 5–6 years. In addition, we showed that the magnitude of the BMI peak explained most of the variance of the body composition measures at 5–6 years. Although the associations between the timing of the BMI peak and the body composition measures at the age of 5–6 years were highly significant, the timing of the BMI peak did not add much information.

Although there were some weak trends in the associations between the BMI peak and blood pressure measures at the age of 5–6 years, the explained variance by the BMI peak was very low. In addition, the significant relation found in boys between the magnitude of the BMI peak and the systolic blood pressure at 5–6 years was possibly caused by the ‘reversal paradox’ [41]. Tu et al. showed that when there is no relation between birth weight and later blood pressure, correcting for current weight (which is positively correlated to birth weight) introduces a negative relation between birth weight and blood pressure. This same phenomenon can be observed in our data, where the magnitude of the BMI peak is positively correlated to current BMI.

However, the BMI peak could have an indirect influence on the blood pressure. The magnitude of the BMI peak and the timing of the BMI peak were both positively associated with the BMI at 5–6 years. In our data the BMI at 5–6 years was significantly positively associated with blood pressure at 5–6 years of age. This strong association between BMI and blood pressure was also found in other studies [40], [42]. In both studies a significant positive effect of BMI on blood pressure was found in both obese and non-obese children. In addition, other overweight indicators such as weight to height [43]–[45] and fat percentage [46] are strongly positively associated with blood pressure. Moreover, in our study birth weight has a negative impact on both blood pressure measures. This phenomenon was also found in other studies [14]–[16].

### Relation to other studies

There have been few studies that have investigated the relation between the BMI peak and later body composition characteristics explicitly [22], [29], [30] and our results are in line with their findings; the outcome at later age, measured with BMI, was strongly significantly associated with the magnitude of the BMI peak. In this study, we also showed that other body composition measures (waist to height ratio and fat percentage) were strongly positively associated with the magnitude of the BMI peak. This is in line with studies indicating that accelerated postnatal growth is associated with an accumulation of fat and higher waist to height ratio [47], [48].

The associations with the health check measures at 5–6 years and the BMI at 9 months of age were comparable (slightly weaker ) to the associations between the health check measures at 5–6 years and the magnitude of the BMI peak. In addition, the BMI peak was strongly correlated (r = 0.925) with the BMI at 9 months of age. This would suggest that the BMI at 9 months of age might be used as a surrogate measure for the magnitude of the BMI peak. This BMI at 9 months has the great advantage that it is easy to obtain by a single measurement, whereas the BMI peak can only be reconstructed with many repeated measurements. More research is required to confirm the validly of using the BMI at 9 months of age as a surrogate measure for the BMI peak.

In the literature, there is evidence that supports an association between early growth patterns and later blood pressure [32]. Especially accelerated growth between 0–6 months increases the risk of hypertension in later stages of childhood [49]–[51]. Although there were no or weak associations between the BMI peak and blood pressure measures at 5–6 years, the combination of the BMI peak and birth weight might be associated with increased blood pressure. Further research is needed to investigate this association.

In our population there was no significant association between birth weight and BMI between 5–6 years of age in the models with the estimated BMI peaks (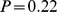 and 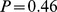 for boys and girls respectively). This is contradictory to the results of Silverwood et al. [23], who found a significant effect of birth weight on the BMI z-score at age 5–13 years . However, univariate analyses of the association between birth weight and the health checks measurements in our ABCD data showed that birth weight was significantly positively associated with BMI between 5–6 years (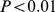 for boys and 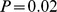 for girls). An explanation for the disappearance of this significant association is that the BMI peak explained part of the effect of birth weight. In our data, there was correlation between birth weight and the magnitude of the BMI peak (r = 0.29 and r = 0.20 for respectively boys and girls).

### Early growth patterns and the adiposity rebound

Our outcome measurements were performed around 5–6 years of age, just before the average timing of the adiposity rebound. Unfortunately, we were not able to reconstruct the adiposity rebound with our data and therefore we cannot make direct inference about the relation between the BMI peak and the adiposity rebound. However, reference growth charts of BMI between the ages 0–20 given by Rolland-Cachera et al. [52] (figure 1) and Cole [26] (figure 1) suggest that the magnitude of the BMI peak is related to the height and timing of the adiposity rebound. In these charts, there is a clear relation between a higher BMI peak and an earlier adiposity rebound.

It is important to stress that we cannot state that our BMI at the health check is indeed the adiposity rebound. Because the adiposity rebound occurs at earlier age for children with a high BMI peak, we might be looking at the point in the BMI curve were the BMI is increasing. For children with a low BMI peak, the opposite could happen and we might be looking at a point in their BMI curve where the BMI is still decreasing.

### Problems of the BMI peak

Estimated BMI peaks were used to quantify the association between the BMI peak and later measures. A potential problem of using estimated BMI peaks was that we did not incorporate the uncertainty of the estimated BMI peaks in our models. Therefore an estimated peak based on, for instance, only 4 BMI measurements between the ages of 0–1 years had a similar weight in our regression analysis as a peak estimated with 10 BMI measurements between the ages of 0–1 years. We also investigated the relation between the BMI peak and later measurements by excluding BMI peaks based on less than four, five, or six measurements, but these analysis gave approximately similar results (data not shown).

Although the BMI peak could be estimated for a large number of children, the estimation failed for 468 children (17%). Especially the timing at which the adiposity peak occurred, was influenced by this phenomenon. In figure 2, a small cluster of BMI peaks was located at the age of 3 months. Close inspection of the BMI curves from children with no BMI peak or a BMI peak showed that most of these children have one or more suspicious BMI measurements. Measurement error or wrong data entry might be a reason for these wrong data points. However, we decided to keep all children with identified peaks in the dataset because the number of children with a suspicious timing of the BMI peak was relatively small.

### Conclusion

We showed that the magnitude of the BMI peak is strongly associated with body composition and, to a lesser extent with blood pressure, at the age of 5–6 years. The absolute value of the BMI at nine months gave essentially the same results. Therefore, the BMI at nine months might be a useful measure in the youth health care practice to identify children at young age with increased risk for obesity and hypertension later in life. However, future research is first required to affirm our findings in children at older ages and to define cut-off values for BMI at nine months with sufficient predictive value. This research is justified as tools to prevent childhood obesity at an early age are urgently needed.

## Supporting Information

Table S1 Associations between BMI at 9 months and body composition measures at 5–6 years of age. Estimated regression parameters, standard errors, and P-values for the linear multivariate regression models describing the relation between the BMI at 9 months, pregnancy duration and the body composition outcomes at the health check. All covariates were centralized around their mean value. The age at outcome measurement was centered around 69 months (coefficients not reported in table)
(PDF)

Table S2 Associations between BMI at 9 months and systolic blood pressure at 5–6 years of age. Estimated regression parameters, standard errors, and P-values for the linear multivariate regression models describing the BMI at 9 months, pregnancy duration and systolic blood pressure outcomes at the health check. All covariates were centralized around their mean value. In addition, the age at outcome measurement was centered around 69 months (coefficients not reported in table).
(PDF)

Table S3 Associations between BMI at 9 months and diastolic blood pressure at 5–6 years of age. Estimated regression parameters, standard errors, and P-values for the linear multivariate regression models describing the relation between the BMI at 9 months, pregnancy duration and diastolic blood pressure outcomes at the health check. All covariates were centralized around their mean value. In addition, the age at outcome measurement was centered around 69 months (coefficients not reported in table).
(PDF)
