# Supplementary material for: Association between Infancy BMI Peak and Body Composition and Blood Pressure at Age 5–6 Years
Source: PLoS One. 2013 Dec 4;8(12):e80517. doi: 10.1371/journal.pone.0080517 (PMC3851737; doi:10.1371/journal.pone.0080517)
Supplement: Table S1 — Associations between BMI at 9 months and body composition measures at 5–6 years of age. Estimated regression parameters, standard errors, and P-values for the linear multivariate regression models describing the relation between the BMI at 9 months, pregnancy duration and the body composition outcomes at the health check. All covariates were centralized around their mean value. The age at outcome measurement was centered around 69 months (coefficients not reported in table) (PDF) [file pone.0080517.s001.pdf]

		Boys			Girls		
Outcome	Covariate	Estimate	Std. Error	P-value	Estimate	Std. Error	P-value
BMI (kg m^{-2})	Intercept	15.497	0.042	—	15.403	0.048	—
	BMI at 9 months (kg m^{-2})	0.498	0.033	< 0.001	0.492	0.034	< 0.001
	Birth weight (kg)	0.300	0.097	0.002	0.352	0.113	0.002
	Pregnancy duration (days)	0.001	0.006	0.839	-0.011	0.006	0.076
Waist to Height ratio	Intercept	45.209	0.094	—	44.608	0.101	—
	BMI at 9 months (kg m^{-2})	0.724	0.073	< 0.001	0.686	0.072	< 0.001
	Birth weight (kg)	-0.139	0.214	0.517	0.179	0.239	0.453
	Pregnancy duration (days)	0.001	0.012	0.948	-0.013	0.013	0.300
Fat percentage (%)	Intercept	19.427	0.203	—	21.335	0.217	—
	BMI at 9 months (kg m^{-2})	1.281	0.159	< 0.001	1.399	0.154	< 0.001
	Birth weight (kg)	1.457	0.462	0.002	0.668	0.512	0.193
	Pregnancy duration (days)	-0.007	0.027	0.796	-0.054	0.027	0.047
