# Supplementary material for: Association between Infancy BMI Peak and Body Composition and Blood Pressure at Age 5–6 Years
Source: PLoS One. 2013 Dec 4;8(12):e80517. doi: 10.1371/journal.pone.0080517 (PMC3851737; doi:10.1371/journal.pone.0080517)
Supplement: Table S2 — Associations between BMI at 9 months and systolic blood pressure at 5–6 years of age. Estimated regression parameters, standard errors, and P-values for the linear multivariate regression models describing the BMI at 9 months, pregnancy duration and systolic blood pressure outcomes at the health check. All covariates were centralized around their mean value. In addition, the age at outcome measurement was centered around 69 months (coefficients not reported in table). (PDF) [file pone.0080517.s002.pdf]

Systolic blood pressure

		Boys			Girls		
Outcome	Covariate	Estimate	Std. Error	P-value	Estimate	Std. Error	P-value
Model 1	Intercept	99.043	0.306	—	98.371	0.294	—
	BMI at 9 months (kg m^{-2})	0.640	0.240	0.008	0.588	0.209	0.005
	Birth weight (kg)	-1.795	0.698	0.010	-0.481	0.694	0.488
	Pregnancy duration (days)	0.034	0.040	0.399	-0.015	0.037	0.678
Model 2	Intercept	99.027	0.301	—	98.318	0.291	—
	BMI at 9 months (kg m^{-2})	0.642	0.236	0.007	0.515	0.208	0.013
	Birth weight (kg)	-2.73	0.715	< 0.001	-1.175	0.706	0.097
	Pregnancy duration (days)	0.046	0.040	0.250	-0.001	0.037	0.974
	Height at outcome measurement (cm)	0.309	0.066	< 0.001	0.266	0.064	< 0.001
Model 3	Intercept	99.015	0.297	—	98.326	0.286	—
	BMI at 9 months (kg m^{-2})	0.053	0.268	0.845	-0.030	0.229	0.895
	Birth weight (kg)	-2.981	0.708	< 0.001	-1.403	0.696	0.044
	Pregnancy duration (days)	0.045	0.039	0.254	0.009	0.036	0.808
	Height at outcome measurement (cm)	0.273	0.065	< 0.001	0.211	0.064	0.001
	BMI at outcome measurement (kg m-2)	1.195	0.270	< 0.001	1.136	0.216	< 0.001
