# Supplementary material for: Association between Infancy BMI Peak and Body Composition and Blood Pressure at Age 5–6 Years
Source: PLoS One. 2013 Dec 4;8(12):e80517. doi: 10.1371/journal.pone.0080517 (PMC3851737; doi:10.1371/journal.pone.0080517)
Supplement: Table S3 — Associations between BMI at 9 months and diastolic blood pressure at 5–6 years of age. Estimated regression parameters, standard errors, and P-values for the linear multivariate regression models describing the relation between the BMI at 9 months, pregnancy duration and diastolic blood pressure outcomes at the health check. All covariates were centralized around their mean value. In addition, the age at outcome measurement was centered around 69 months (coefficients not reported in table). (PDF) [file pone.0080517.s003.pdf]

Diastolic blood pressure

		Boys			Girls		
Outcome	Covariate	Estimate	Std. Error	P-value	Estimate	Std. Error	P-value
Model 1	Intercept	59.085	0.311	—	60.227	0.285	—
	BMI at 9 months (kg m^{-2})	0.344	0.244	0.158	0.221	0.203	0.275
	Birth weight (kg)	-1.007	0.709	0.156	-1.657	0.673	0.014
	Pregnancy duration (days)	0.047	0.041	0.246	0.031	0.036	0.386
Model 2	Intercept	59.078	0.31	—	60.205	0.284	—
	BMI at 9 months (kg m^{-2})	0.345	0.243	0.156	0.191	0.203	0.348
	Birth weight (kg)	-1.392	0.736	0.059	-1.950	0.691	0.005
	Pregnancy duration (days)	0.052	0.041	0.202	0.037	0.036	0.303
	Height at outcome measurement (cm)	0.127	0.068	0.060	0.112	0.062	0.072
Model 3	Intercept	59.07	0.308	—	60.212	0.281	—
	BMI at 9 months (kg m^{-2})	-0.041	0.279	0.882	-0.272	0.225	0.228
	Birth weight (kg)	-1.557	0.735	0.035	-2.144	0.684	0.002
	Pregnancy duration (days)	0.052	0.041	0.205	0.045	0.035	0.200
	Height at outcome measurement (cm)	0.103	0.068	0.128	0.066	0.063	0.294
	BMI at outcome measurement (kg m-2)	0.784	0.281	0.005	0.963	0.212	< 0.001
